# Cathepsin B Deficiency Improves Memory Deficits and Reduces Amyloid-β in hAβPP Mouse Models Representing the Major Sporadic Alzheimer’s Disease Condition

**DOI:** 10.3233/JAD-221005

**Published:** 2023-05-02

**Authors:** Gregory Hook, Mark Kindy, Vivian Hook

**Affiliations:** a American Life Science Pharmaceuticals, La Jolla, CA, USA; b Department of Pharmaceutical Sciences, Taneja College of Pharmacy, University of South Florida, Tampa, FL, USA; c James A Haley VAMC, Research Service, Tampa, FL, USA; d Department of Neuroscience, Department of Pharmacology, Skaggs School of Pharmacy and Pharmaceutical Sciences, University of California, San Diego, La Jolla, CA, USA

**Keywords:** Alzheimer’s disease, amyloid-β, AβPP isoform, β-secretase, cathepsin B, cDNA, gene, memory deficits, mouse models, neuron, promoter

## Abstract

The lysosomal cysteine protease cathepsin B (CTSB) has been suggested as a biomarker for Alzheimer’s disease (AD) because elevated serum CTSB in AD patients has been found to correlate with cognitive dysfunction. Furthermore, *CTSB* gene knockout (KO) in non-transgenic and transgenic AD animal models showed that elimination of CTSB improved memory deficits. However, conflicting CTSB KO results on amyloid-β (Aβ) pathology in transgenic AD models have been reported. The conflict is resolved here as likely being due to the different hAβPP transgenes used in the different AD mouse models. *CTSB* gene KO reduced wild-type (Wt) β-secretase activity, brain Aβ, pyroglutamate-Aβ, amyloid plaque, and memory deficits in models that used cDNA transgenes expressing hAβPP isoform 695. But in models that used mutated mini transgenes expressing hAβPP isoforms 751 and 770, *CTSB* KO had no effect on Wt β-secretase activity and slightly increased brain Aβ. All models expressed the AβPP transgenes in neurons. These conflicting results in Wt β-secretase activity models can be explained by hAβPP isoform specific cellular expression, proteolysis, and subcellular processing. *CTSB* KO had no effect on Swedish mutant (Swe) β-secretase activity in hAβPP695 and hAβPP751/770 models. Different proteolytic sensitivities for hAβPP with Wt versus Swe β-secretase site sequences may explain the different CTSB β-secretase effects in hAβPP695 models. But since the vast majority of sporadic AD patients have Wt β-secretase activity, the CTSB effects on Swe β-secretase activity are of little importance to the general AD population. As neurons naturally produce and process hAβPP isoform 695 and not the 751 and 770 isoforms, only the hAβPP695 Wt models mimic the natural neuronal hAβPP processing and Aβ production occurring in most AD patients. Significantly, these *CTSB* KO findings in the hAβPP695 Wt models demonstrate that CTSB participates in memory deficits and production of pyroglutamate-Aβ (pyroglu-Aβ), which provide rationale for future investigation of CTSB inhibitors in AD therapeutics development.

## INTRODUCTION

Evidence for participation of the lysosomal cysteine protease cathepsin B (CTSB) in Alzheimer’s disease (AD) memory deficits has been provided by human clinical studies. In AD patients, increases in serum CTSB correlate with the extent of cognitive dysfunction [1]. CTSB is also elevated in brain [2] and cerebrospinal fluid (CSF) [3–5] of AD patients. Notably, CTSB accumulates with amyloid plaques in human AD brain [6].

These human AD findings of CTSB upregulation have been investigated in AD animal models by CTSB gene knockout, which demonstrate that CTSB participates in memory deficits [7, 8]. Studies have also assessed amyloid-β (Aβ) in several variant human amyloid-β protein precursor (hAβPP) transgenic mouse models with conflicting results regarding Aβ production or degradation by cathepsin B. Therefore, the purpose of this review is to provide detailed analysis of the compiled CTSB data to understand its role in AD with respect to 1) elevation of CTSB in AD patients with the major sporadic population, 2) gene knockout of CTSB in AD animal models resulting in improved memory deficits, 3) participation of CTSB in Aβ production in hAβPP models with wild-type (Wt) β-secretase site representing the major sporadic AD population, but not in hAβPP models with the Swe mutant β-secretase site representing a minor portion of AD, and 4) the overall consilience of data showing participation of CTSB in memory deficits and Aβ production in hAβPP models representing the major sporadic AD population.

## ELEVATION OF CTSB IN AD PATIENTS

Numerous studies have demonstrated increased levels of CTSB in AD patients (Table 1). CTSB protein in the temporal cortex of human AD brains was increased by 80% compared to age-matched controls [2]. CTSB has been shown to accumulate in amyloid plaques in human AD brains [6]. CTSB protein levels in serum and plasma increased by 50% compared to controls (age-matched) [1, 4]. Significantly, high CTSB levels in serum were strongly correlated with cognitive decline in AD patients [1]. Also, in CSF, CTSB protein levels were greater in AD patients compared to controls by proteomics and western blot assessments [3–5]. In chronic periodontitis-associated AD patients, CTSB in serum was higher than controls by 43% [9]. Notably, the higher levels of serum CTSB corelated with reduced Mini-Mental State Examination scores of cognitive function in these periodontitis AD patients [9]. These findings demonstrate upregulation of CTSB in brain and peripheral serum or plasma of AD patients.

**Table 1:** Elevation of cathepsin B in Alzheimer’s disease patients

Clinical	Biofluid or	CTSB	Features	Reference
Condition	Tissue	Regulation
AD	brain cortex	↑	CTSB protein increased by 18-fold	[2]
AD	brain	↑	High CTSB protein and proteolytic activity abnormally localized at amyloid plaques in brain	[6]
AD	serum	↑	increased CTSB correlated with cognitive deficits	[1]
AD	CSF	↑	increased CTSB protein	[4, 5]
AD	CSF	↑	Increased CTSB protein in AD analyzed by proteomics	[3]
AD	plasma	↑	elevated CTSB protein in mild and severe AD by 50–80% above controls	[70]
Periodontitis	serum	↑	increased CTSB levels by 43%	[9]
associated AD

Studies showing increased cathepsin B (CTSB) levels in brain, cerebrospinal fluid (CSF), and serum or plasma of blood samples from Alzheimer’s disease (AD) patients are indicated by this table.

## CTSB GENE KNOCKOUT RESULTS IN IMPROVED MEMORY DEFICITS IN HUMAN AβPP ANIMAL MODELS OF AD

CTSB deficiency by gene knockout (KO) resulted in improved memory deficits in a transgenic AD model expressing hAβPP-695 [7] and in a non-transgenic chronic-periodontitis-associated AD model [8]. Improved memory deficits were associated with reduced Aβ levels by *CTSB* KO.

### CTSB KO in the hAβPP-695/Wtβ-Lonγ model of AD improves memory deficits

The hAβPP model of AD, expressing hAβPP-695/Wtβ-Lonγ, displays memory deficits and amyloid plaque brain pathology [7]. *CTSB* KO in these AD mice resulted in significant improvement in memory deficits to nearly normal memory function, assessed by the Morris water maze test [7]. Improved memory by *CTSB* KO was also indicated by the increased time that mice spent in the quadrant with the submerged platform that the mice were trained to recall. Amyloid plaque pathology was significantly decreased by *CTSB* KO in these AD mice and was accompanied by decreased brain levels of Aβ_1 - 40_, Aβ_1 - 42_, pGlu-Aβ_3 - 40_, and pGlu-Aβ_3 - 42_ peptides [7, 10]. These *CTSB* KO data indicate participation of CTSB in AD memory deficits and production of Aβ peptides [7].

In contrast, *CTSB* KO in a Swedish (Swe) mutant AD model expressing hAβPP-695/Sweβ-Lonγ had no effect on memory deficits [7]. The Swe mutant represents a minor portion of AD patients from one family [11]. *CTSB* KO also had no effect on Aβ peptide levels in the hAβPP-695/Sweβ-Lonγ mice.

These combined *CTSB* KO data in different hAβPP models are consistent with the hypothesis that CTSB participates in regulating Aβ produced from hAβPP with the Wt β-secretase site representing the major sporadic AD population, but not from hAβPP with the Swe mutant β-secretase site that represents a minor percentage of AD. Further data in the field of hAβPP models with the Wt or Swe mutant β-secretase sites in *CTSB* KO studies is discussed in this review.

### CTSB KO in chronic periodontitis-associated AD model improves memory deficits

Infection by *Prophyromonas gingivalis*, the major periodontal bacteria, has been shown to be positively linked to AD and cognitive dysfunction [12, 13]. *Prophyromonas gingivalis* lipopolysacharide (PgLPS) has been found in human AD brain [14]. Notably, cognitive deficits of periodontitis patients correlate with increased levels of cathepsin B [9]. Therefore, cathepsin B knockout in a mouse model of periodontitis was assessed for predicted improvements in memory deficits [8]. Significant results showed that CTSB participates in PgLPS-induced periodontitis and memory deficits [8]. KO of the *CTSB* gene in the periodontitis model of AD (PgLPS mice) resulted in significant improvements in memory deficits in middle-aged mice of 12 months old, but not in young 2-month-old mice, treated with PgLPS for 5 weeks. The PgLPS induction of CTSB in brain hippocampus was consistent with its blockade by *CTSB* gene KO that alleviated memory deficits. *CTSB* KO blocked PgLPS-induced activation of the inflammatory factors IL-1β and toll-like receptor 2. *CTSB* KO also blocked PgLPS-induced increases in Aβ_42_ in brain. These data illustrate participation of CTSB in memory deficits, inflammation, and Aβ production from Wt AβPP in the PgLPS model of periodontitis-associated AD memory deficits [8].

## Aβ REGULATION BY CTSB KO IN hAβPP MOUSE MODELS: CONFLICTING DATA EXPLAINED BY VARIANT hAβPP ISOFORM MODELS, TRANSGENES, NEURONAL VERSUS GLIA EXPRESSION, AND WT OR SWE MUTANT β-SECRETASE SITE OF hAβPP

*CTSB* KO studies in AD mouse models have investigated regulation of Aβ [7, 10, 15–17]. The Hook group showed that CTSB participates in Aβ production, shown by reduced Aβ levels in *CTSB* KO AD mice compared to controls [7, 10, 15]. However, the Gan group suggests that CTSB participates in Aβ degradation, shown by small increases in Aβ in CTSB KO mice in different AβPP models [16, 17].

These results by the Hook and the Gan groups appear to be conflicting, but the different findings can be explained by differences in hAβPP isoforms expressed in the mouse models. These models differ in hAβPP-695 or hAβPP-751/770 isoforms expressed, transgenes, alternative RNA splicing that generates hAβPP isoforms, neuronal compared to glia expression of hAβPP, and Wt compared to Swe mutant β-secretase sites of hAβPP isoforms.

To provide the field with an understanding of these different hAβPP models, detailed analysis of these variant hAβPP models is explained in the next section “AD models expressing variant human AβPP isoforms.” Then, evaluations are provided for *CTSB* KO in Wt β-secretase site hAβPP models representing the major sporadic AD population [18], and *CTSB* KO in Swe mutant β-secretase site hAβPP models which represent a minor portion of AD patients in one family [19, 20]. Overall, data from hAβPP models representing the major AD sporadic condition show that *CTSB* KO results in decreased levels of brain Aβ peptides.

### AD models expressing variant hAβPP isoforms

The six different hAβPP models of AD used in *CTSB* KO studies differed in hAβPP isoforms, gene constructs, promoter driven expression for natural or abnormal expression of hAβPP isoforms in neurons compared to glia, and hAβPP containing Wt or Swe mutant β-secretase sites. These features of the hAβPP mouse models are summarized in Table 2. These models consisted of four different hAβPP-695 isoform mouse models used by the Hook group [7, 10, 15], and two variant hAβPP-751/770 models used by the Gan group [16, 17]. Details of the different features of these variant hAβPP models are provided in the following sections.

#### Normal hAβPP gene transcription and alternative splicing generates hAβPP isoforms of 695, 751, and 770 residues

**Fig. 1:**
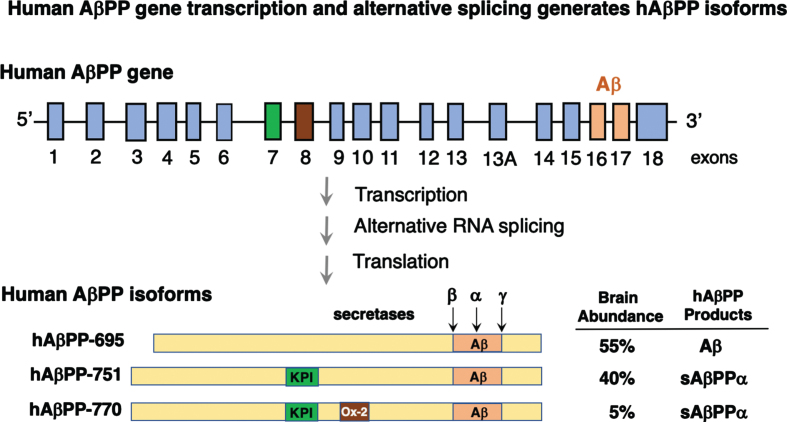
Human AβPP gene transcription and alternative splicing generates AβPP isoforms. The hAβPP gene structure consists of 18 exons with introns [23]. The Aβ, KPI (kunitz protease inhibitor), and Ox-2 domains are encoded by exons 16-17, exon 7, and exon 8, respectively. Alternative RNA splicing of the gene transcript generates three main hAβPP isoforms of hAβPP-695, hAβPP-751, and hAβPP-770 that all contain the Aβ domain. The hAβPP-751 and hAβPP-770 isoforms contain the KPI domain. The hAβPP-770 isoform includes the Ox-2 domain. In brain, hAβPP-695 is the most abundant isoform and is present in neurons for production of amyloidogenic Aβ by β-secretase and γ-secretase [22–24]. The hAβPP-751 and hAβPP-770 isoforms are present at low levels in brain [21, 23–26], and are converted to non-amyloidogenic sAβPPαby *α*-secretase cleavage [27, 38].

The hAβPP gene is composed of 18 exons that undergo alternative splicing to generate isoforms of hAβPP-695, hAβPP-751, and hAβPP-770 (Fig. 1). These three hAβPP isoforms differ in their amino acid lengths of 695, 751, and 770 residues, which are expressed in brain at approximate relative ratios of 20:10:1 [21] (Fig. 1). All hAβPP isoforms contain the Aβ domain. The hAβPP-751 and hAβPP-770 isoforms contain the kunitz protease inhibitor (KPI) domain. The hAβPP-770 isoforms also include the Ox-2 domain.

Human AβPP-695 is the most abundant isoform present in brains of AD and normal conditions (∼65% of total hAβPP) and is exclusively expressed in neurons where it is processed into amyloidogenic Aβ peptides [22–24].

The hAβPP-751 and hAβPP-770 isoforms are expressed at low levels in brain compared to hAβPP-695 [21, 24]. AβPP-751 and AβPP-770 are expressed in glia cells where they undergo processing to generate non-amyloidogenic sAβPPα[21, 24–28].

#### Different hAβPP isoforms and transgene constructs of AD models used in CTSB KO studies by the Hook group compared to the Gan group

**Fig. 2:**
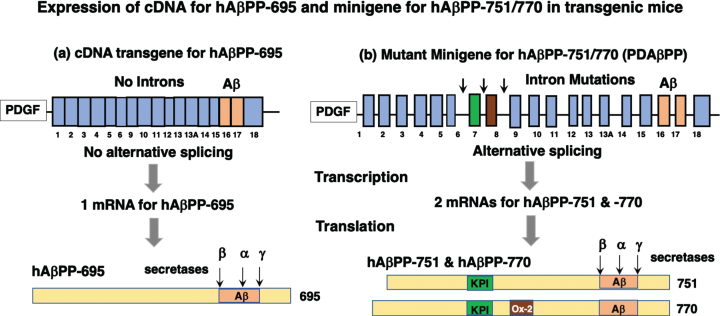
Human AβPP cDNA and minigene expression of hAβPP-695 and hAβPP-751/770 isoforms. (a) cDNA expression of hAβPP-695. The Hook group expressed the cDNA of hAβPP-695 in mouse studies of CTSB KO [7, 10, 15]. (b) Mutant minigene expression of hAβPP-751/770. In contrast, the Gan group expressed a minigene of hAβPP-751/770 with mutations in introns between exons 6 and 9 (indicated by arrows) [16, 17, 23]. The minigene produced multiple RNAs, underwent alternative splicing, and produced 45.8% hAβPP-770, 46.7% hAβPP-751 and 7.5% hAβPP-695 [23]. But normal brain produces much higher levels of hAβPP-695 than hAβPP-751/770 [21, 24].

AD models used by the Hook and Gan groups differ in hAβPP isoforms expressed (Fig. 2). The Hook group utilized hAβPP-695 models expressing the cDNA of hAβPP-695 for direct mRNA expression and protein translation, without RNA splicing (Fig. 2a) [7, 10, 15]. This model represents hAβPP-695 as the major hAβPP isoform in brain [21, 24].

In contrast, the Gan group expressed a mutant minigene construct of hAβPP-751/770 (called PDAβPP) containing an engineered gene whereby the three introns between exons 6 and 9 were mutated [16, 17, 23] (Fig. 2b). These mutations consisted of a 1,515 base pair deletion in the intron between exons 6 and 7 and a restriction endonuclease site was engineered. In the intron between exons 7 and 8, a restriction endonuclease site was eliminated and four unspecified base pairs were added. In the intron between exons 8 and 9, 2,066 base pairs were deleted. The minigene produced multiple RNAs, underwent alternative splicing, and produced 45.8% hAβPP-770, 46.7% hAβPP-751, and 7.5% hAβPP-695 [23]. As such, the mutant minigene produced abundant levels of hAβPP-751 and very little hAβPP-695, which does not represent brain hAβPP isoform levels (Fig. 1).

#### Normal expression of endogenous hAβPP isoforms in neurons and glia cells, normal PDGF transgene expression of hAβPP-695 in neurons, and abnormal PDGF transgene expression of hAβPP-751/770 in neurons

**Fig. 3:**
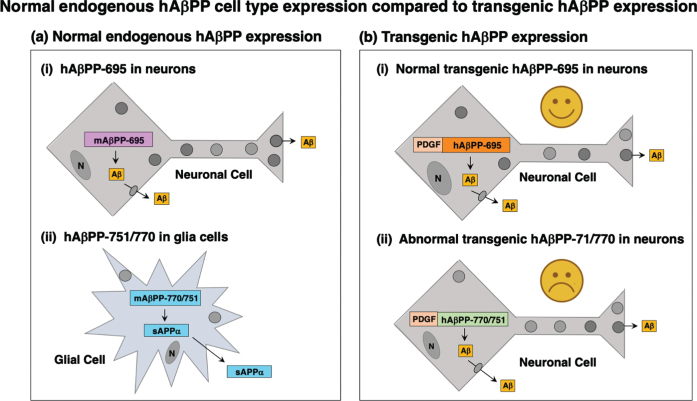
Normal hAβPP-695 expression in neurons and hAβPP-751/770 in glia cells, but abnormal hAβPP-751/770 transgene expression in neurons. (a) Normal endogenous expression of hAβPP isoforms. The hAβPP-695 isoform is exclusively expressed in neurons for Aβ production [22–24], and the hAβPP-751/770 isoforms are normally expressed in glia cells [27, 28]. (b) Transgenic expression of hAβPP-695 in its normal neuronal cell type, but abnormal expression of hAβPP-751/770 in neurons. Expression of the hAβPP-695 under the control of the PDGF promoter results in expression in neurons, the normal cell type for this isoform as conducted by the Hook group [7, 10, 15]. But PDGF driven expression of hAβPP-751/770 results in abnormal expression in neurons [16, 17, 23], rather than in the normal location of glia cells [27, 28].

Endogenous hAβPP-695 isoform is exclusively expressed in neurons and is processed into Aβ [22–24] (Fig. 3a.i). In contrast, endogenous hAβPP-751 and hAβPP-770 isoforms are expressed primarily in glia cells and generate nonamyloidogenic sAβPPα[27, 28] (Fig. 3a.ii).

In the hAβPP transgenes used by the Hook group (Table 2) [7, 10, 15] the PDGF promoter drives neuronal expression of hAβPP-695 (Fig. 3b.i) which represents its normal endogenous neuronal cell localization (Fig. 3a.i). Thus, the four PDGF hAβPP-695 models used by the Hook group (listed in Table 2) study normal hAβPP-695 expression and function in neurons.

However, the PDGF promoter in the minigene used by the Gan group (Table 2) resulted in abnormal neuronal expression of hAβPP-751/770 (Fig. 3b.ii) as these isoforms are normally expressed in glia cells (Fig. 3a.ii). Thus, the PDGF hAβPP-751/770 models (listed in Table 2) represent abnormal expression of hAβPP-751/770 in neurons. The processing of hAβPP-751/770 in the abnormal neuronal location will likely differ from glia cells, since each cell type has its distinct trafficking and proteolytic systems.

#### Human AβPP models with the Wt β-secretase site represent the major sporadic AD population, while hAβPP isoforms with the Swe mutant β-secretase site represent a small number of AD patients from one family

Models expressing hAβPP result in its proteolytic processing to generate Aβ in brain. Aβ is generated from AβPP by proteolytic cleavage at the β-secretase and the γ-secretase sites that flank Aβ (Fig. 1).

The Wt β-secretase of hAβPP is expressed by the major sporadic AD population [18]. Thus, models expressing hAβPP with the Wt β-secretase site are important to gain understanding of the major sporadic AD population possessing no known mutations.

The Swe mutant hAβPP is expressed in one AD family and represents a very small number of AD patients [11]. Studies of the Swe mutation of hAβPP in mouse models have been of interest to understand mechanisms of elevated Aβ and impaired memory that occurs in AD. The Swe hAβPP mutation is relevant to the AD family possessing this inherited genetic mutation associated with AD [11].

Models utilized in CTSB KO studies consisted of those expressing hAβPP-695 and hAβPP-751/770 with the Wt and the Swe mutant β-secretase sites (Table 2). Human AβPP models with the Wt β-secretase site provide analysis of Aβ production by Wt β-secretase activity. The Wt β-secretase activity represents the majority of the AD population.

#### Models with γ-secretase mutations of hAβPP display amyloid plaques and memory deficits

Human AβPP contains the γ-secretase site sequence which is cleaved after β-secretase cleavage by the γ-secretase complex to produce Aβ. While most AD patients express hAβPP with the Wt γ-sequence, familial mutations near this site occurs and hAβPP models with such familial γ-sequence site mutations overproduce Aβ and develop amyloid plaque with memory deficits but have Wt β-secretase activity. These models provide assessment of Aβ, amyloid plaques, and memory deficits.

Studies of CTSB KO were conducted in mice expressing Wt hAβPP-695 [15], or hAβPP-695/Wtβ with Lon (V717I) [7] or Ind (V717F) [10] γ-secretase mutations (Table 2). Studies have also used mice expressing hAβPP-751/770 with Wt or the Ind γ-secretase site mutation of hAβPP [16, 17] (Table 2).

### CTSB KO in the hAβPP-695 model with WT β-secretase site reduced Aβ, but CTSB KO in the hAβPP-751/770 model with Wt β-secretase site resulted in a small elevation of Aβ

*CTSB* KO in hAβPP-695 AD mice substantially reduced Aβ [7, 10, 15], but CTSB KO in hAβPP-751/770 AD mice resulted in a slight increase in Aβ [16, 17]. These different Aβ results can be explained by use of different hAβPP isoform models.

#### CTSB KO reduced Aβ and amyloid plaques in the hAβPP-695/Wtβ-Lonγ AD mice

In the hAβPP-695/Wtβ-Lonγ AD model, *CTSB* KO substantially reduced Aβ peptide levels and amyloid plaque pathology [7, 10]. Absence of CTSB resulted in decreased brain Aβ_40_ and Aβ_42_ by ∼85% and reduced amyloid plaques by ∼85%. *CTSB* KO reduced the β-secretase product CTFβ by 60% and increased the non-amyloidogenic product sAβPPαby 60% [7]. CTFβ and sAβPPαare biomarkers of β-secretase activity; their changes that occurred in the *CTSB* KO condition indicate reduced β-secretase activity. Furthermore, CTSB expression increased brain levels of Aβ_40_ and Aβ_42_ by 150% and 200%, respectively. compared to controls of 100% [10]. These data demonstrate participation of CTSB in the upregulation of β-secretase activity for Aβ production.

Additional evidence for *CTSB* KO reduction of Aβ was demonstrated in mice expressing Wt hAβPP695 having no mutations (hAβPP695Wt) [15]. The absence of CTSB resulted in reduced human Aβ_40_ and Aβ_42_ by 70%, reduced CTFβ by 40%, and increased sAβPPαby 160% compared to controls of 100% [15]. These results provided further support for CTSB participation in Aβ production in brain.

Human AD brains contain elevated levels of pyroglutamate-modified pGlu-Aβ_3 - 40_ and pGlu-Aβ_3 - 42_ forms of truncated Aβ whose high toxicity occurs by promoting aggregation of Aβ peptides [29–31]. In the hAβPP-695/Wtβ-Lonγ AD mice, *CTSB* KO reduced pGlu-Aβ_3 - 40_ by 65%, reduced pGlu-Aβ_3 - 42_ by 90%, also reduced pGlu-Aβ amyloid plaque by 46% in brain [10]. Furthermore, overexpression of CTSB increased pGlu-Aβ_3 - 40_ and pGlu-Aβ_3 - 42_ by 150% and 200% compared to controls of 100%, with increased pGlu amyloid plaque load by 178%. These data demonstrate participation of CTSB in producing pGlu-modified Aβ peptides.

#### CTSB KO in the hAβPP-751/770/Wt model resulted in no change in hippocampal Aβ and a small elevation in cortical Aβ in mouse brain

While *CTSB* KO in human AβPP-695/Wtβ-Lonγ AD mice resulted in lowered Aβ species [7, 10, 15], *CTSB* KO in hAβPP-751/770/Wt mice had no effect on hippocampal Aβ_42_ and increased Aβ_1 - x_ by 18% above controls, and resulted in small increases in Aβ_42_ and Aβ_1 - x_ in brain cortex of 20% and 24% above controls [17]. Further studies examined consequences of elevating CTSB by overexpression or by deleting cystatin C, an endogenous inhibitor of CTSB. These conditions of increased CTSB in hAβPP-751/770Wt mice resulted in no change in Aβ_1 - x_ and decreased Aβ_42_ by 12% in hippocampus compared to controls. Increased CTSB had no effect on levels of CTFβ, CTF*α*, and sAβPP*α*. These data in hAβPP-751/770 mice suggest in these conditions, CTSB may be involved in degradation of brain Aβ [17].

Significantly, memory function was not assessed [17] and, thus, findings of the relationship of small increases in Aβ resulting from CTSB KO with memory function are unknown.

#### Different hAβPP isoforms explain the apparently conflicting results of CTSB KO reduction of Aβ in the hAβPP-695/Wtβ-Lonγ and hAβPP695Wt models versus the hAβPP-751/770/Wt model

The PDGF hAβPP-695/Wtβ-Lonγ model expressed hAβPP-695 in neurons [7], mimicking the *in vivo* neuronal expression of the major hAβPP-695 isoform in brain (Fig. 3). The exclusive expression of hAβPP-695 in neurons yields production of amyloidogenic Aβ peptides [21, 23, 25, 26, 28].

In contrast, the hAβPP-751/770/Wt model [17] resulted in abnormal expression of hAβPP-751/770 in neurons (driven by the PDGF promoter) which did not represent the normal endogenous glia cell expression of hAβPP-751/770 (Fig. 3). Normal glia expression of hAβPP751/770 produces non-amyloidogenic sAβPPα[22, 27, 28]. Furthermore, hAβPP-751/770 are minor isoforms of hAβPP in brain [21, 23, 25, 26, 28]. Results show that the abnormal hAβPP-751/770/Wt model can produce low amounts Aβ that is independent of CTSB.

It is important to utilize the model that best represents the normal production of Aβ in neurons from hAβPP-695. Therefore, the hAβPP-695/Wtβ-Lonγ model logically represents normal endogenous production of Aβ in neurons involving CTSB.

### CTSB KO in hAβPP-695 mice with Swe mutant β-secretase site had no effect on Aβ, but CTSB KO in hAβPP-751/770 mice with Swe mutation resulted in a small elevation of Aβ

As discussed above, CTSB KO in hAβPP-695 models with the Wt β-secretase site resulted in reduced Aβ and reduced Wt β-secretase activity [7, 10, 15]. But in hAβPP-695 models with the Swe mutant β-secretase site, *CTSB* KO had no effect on Aβ levels [8, 15]. These different results may be due to CTSB having or effecting cleavage of the Wt β-secretase site, but not the Swe β-secretase site.

In the hAβPP-751/770 model with the Swe mutant β-secretase site, *CTSB* KO resulted in a small elevation of Aβ [16]. The reason for the no effect versus slight increase in Aβ between the Swe mutant hAβPP-695 models versus Swe mutant hAβPP-751/770 is unclear but may result from the abnormal neuronal hAβPP-751/770 expression.

#### CTSB KO in hAβPP-695/Sweβ-Lonγ AD mice had no effect on Aβ or Swe mutant β-secretase activity

The Swe mutation consists of Asn-Leu instead of the normal Lys-Met amino acid sequence at the β-secretase site of hAβPP [11]. Studies of *CTSB* KO in Swe mutant hAβPP mice expressing hAβPP-695/Sweβ-Lonγ, which mimicked normal neuronal expression, showed no effects on Aβ_40_, Aβ_42_, CTFβ, sAβPP*α*, and memory deficits [7]. These data showed that CTSB was not involved in production of Aβ from Swe mutant β-secretase activity. However, CTSB participates in Aβ production from hAβPP-695 having the Wt β-secretase site and utilizing Wt β-secretase activity [7].

#### CTSB KO in hAβPP-751/770/Sweβ-Indγ AD mice had no effect on Swe mutant β-secretase activity and produced a small increase in Aβ and amyloid plaque

Studies of mice expressing hAβPP-751/770Sweβ-Indγ, representing abnormal neuronal rather than glia expression, showed that *CTSB* KO had no effect on CTFβ levels in brain which indicates no effect on Swe β-secretase activity [16]. These findings are consistent with *CTSB* KO having no Swe β-secretase activity in mice expressing hAβPP-695/Sweβ-Lonγ [7]. However, KO of CTSB in the hAβPP-751/770Sweβ-Indγ mice resulted in a small increase in the ratio of Aβ_42_/Aβ_1 - x_ and increased amyloid plaque, suggesting that CTSB may be involved in degradation of Aβ [16]. Lentiviral CTSB expression reduced preexisting amyloid deposits, also suggesting CTSB degradation of Aβ.

#### CTSB cleaves the Wt β-secretase site but not the Swe mutant β-secretase site

*CTSB* KO data supports participation of CTSB in regulating Wt β-secretase activity to generate Aβ peptides but CTSB does not participate in Swe mutant β-secretase activity in Aβ production (Table 2). To test the hypothesis that CTSB may function as an alternative β-secretase, CTSB cleavage of the Wt β-secretase site of the model Z-Val-Lys-Met-↓AMC substrate was assessed. CTSB has high activity for cleaving the Wt β-secretase site (Table 3) [32]. However, CTSB showed almost no cleavage of the Swe mutant β-secretase site of the Z-Val-Asn-Leu-↓AMC (Asn-Leu is the Swe mutation) substrate (Table 3) [32]. CTSB displayed a 2,735-fold higher rate of cleaving the Wt over the Swe mutant substrates (Table 3). CTSB clearly prefers to cleave the Wt Z-Val-Lys-Met-↓AMC substrate compared to the Swe mutant substrate Z-Val-Asn-Leu-↓AMC. These results demonstrate CTSB as an alternative Wt β-secretase in addition to the well-established BACE1 β-secretase [33–35].

**Table 2:** Human AβPP animal models used in CTSB KO studies for Aβ evaluation

Human AβPP	Human AβPP Model Features				CTSB KO Improves Memory Deficits			References
Model					and Modulates Aβ		
	AβPP type, promoter	DNA form	β-Secretase site	γ-Secretase site	Memory Deficits	Aβ Biomarkers	Aβ Pathology
hAβPP-695/Wt	**hAβPP-695:** PDGF neuronal	cDNA	WT	WT	n/a	↓ Aβ_1 - 42_ by ∼70% ↓ Aβ_1 - 40_ by ∼70% ↓CTFβ by 40%, ↑ sAβPPα by 60%, ↓WT β-secretase activity	n/a	[15]
hAβPP-695/Wtβ-Lonγ	**hAβPP-695:** PDGF neuronal	cDNA	WT	Lon	↓Memory deficits	↓Aβ_1 - 40_ by 85% ↓Aβ_1 - 42_ by 87% ↓pGluAβ_3 - 40_ by 65% ↓pGlu Aβ_3 - 42_ by 92% ↓CTFβ by 60% ↑ sAβPPα by 60% ↓WT β-secretase activity	↓Aβ plaque by 85%, ↓pGluAβ plaque by 46%	[7, 10]
hAβPP-695/Sweβ-Lonγ	**hAβPP-695**: PDGF neuronal	cDNA	Swe	Lon	no effect on memory deficits	no effects on Aβ_1 - 42_, CTFβ, or AβPP*α*	no effect on amyloid plaque	[7]
hAβPP-695/Sweβ-Indγ	**hAβPP-695,** PDGF neuronal	cDNA	Swe	Ind	n/a	No effects on Aβ, CTFβ, sAβPP*α*	n/a	[15]
hAβPP-751/770/Sweβ-Indγ	**hAβPP-Swe-Ind-751/770,** PDGF neuronal (J20 line, introns modified, PDAPP)	minigene	Swe	Ind	nd	no change in flAβPP, CTFβ, *α*-sAβPP, *α*-CTF ↑ Aβ_1 - 42_/Aβ_1 - x_ ratio by ∼25%	elevated plaque load	[16]
hAβPP-751/770/Wt	**hAβPP-751/770,** PDGF neuronal (I63 line, introns modified, PDAPP)	minigene	WT	WT	nd	no change in hippocampal Aβ_42_, ↑ cortical Aβ_42_ by 12%	nd	[17]

All human AβPP models utilized C57BL/6 mouse strain (adult ages 3–12 months) using equal numbers of male and female mice. Swe, K670N/M671L/Lon V717I/Ind V717F; nd, not determined; n/a. not applicable.

**Table 3:** Cathepsin B selectively cleaves the WT β-secretase site compared to the Swe mutant site

	WT β-secretase site substrate:	Swe mutant β-secretase site:
	Z-Val-Lys-Met-↓AMC	Z-Val-Asn-Leu-↓AMC
Cathepsin B	100%	0.04%

Cathepsin B cleavage of the WT (wild-type) β-secretase site substrate was compared to the Swe (Swedish) mutant β-secretase site substrate, normalized to cathepsin B proteolytic activity with Z-Val-Lys-Met-↓AMC as 100% [32]. These Z-peptide-AMC model substrates mimic the β-secretase cleavage site within the amyloid-β protein precursor (AβPP).

## CHRONIC PERIODONTITIS-ASSOCIATED AD AND NEURODEGENERATION MODELS HAVE SHOWN CTSB PARTICIPATION IN Aβ PRODUCTION VIA WT β-SECRETASE

Models of chronic periodontitis-associated AD, advanced glycation end (AGE) products, and Mucopolysaccharidosis type I (MPSI) described in this section provide evidence for participation of CTSB in Aβ production in AD-related neurodegenerative conditions.

### Chronic periodontitis-associated AD

Clinical evidence indicates a positive link between periodontitis and AD with respect to cognitive dysfunction and inflammation [12, 13]. *CTSB* KO in the neuroinflammatory periodontitis model of AD showed that CTSB participates in neuronal Aβ production and drives memory deficits [8]. CTSB KO blocked PgLPS-induced elevation of Aβ_42_ in mouse brain, indicating that Aβ_42_ production is dependent on CTSB [8]. These data show that CTSB regulates Wt β-secretase activity for conversion of mouse AβPP to Aβ. These mouse studies are relevant to clinical periodontitis, since periodontitis patients display elevated serum CTSB that correlates with cognitive deficits [8, 9]. In cellular neuroblastoma studies, inhibition of CTSB with the selective inhibitor CA-074Me reduced PgLPS-induced increases in Aβ_40_ and Aβ_42_ [9]. These results demonstrate participation of CTSB in Aβ production generated from AβPP by Wt β-secretase activity.

### AGE products in aging and neurodegeneration

AGE involves reaction of glucose or other sugars with proteins that induce neuronal toxicity through the AGE receptor [2]. In cortical neurons, AGE increased CTSB and Aβ_42_; furthermore, the cathepsin B inhibitor reduced Aβ_42_. These results indicate that CTSB participates in Aβ_42_ production [2].

### Mucopolysaccharidosis type I (MPS I)

MPS I is a rare neurologic disease resulting from a genetic deficiency of *α*-L-iduronidase (IDUA) involving impaired lysosomal catabolism and neurodegeneration [36]. The MPS I mouse model, generated by KO of the *IDUA* gene, displays increased levels of CTSB and elevated Aβ in brain. The study indicated that CTSB provides an alternative amyloidogenic pathway for Aβ production [36].

### Evidence for CTSB as an alternative Wt β-secretase for Aβ production

Overall, the studies described here have demonstrated a role for CTSB in Aβ production from AβPP having the Wt β-secretase site, indicating CTSB involvement in Wt β-secretase activity. CTSB participates in Aβ production in neurodegenerative disease models of periodontitis, AGE, and MPS I expressing Wt AβPP [8, 2, 36]. Evidence supports CTSB as an alternative Wt β-secretase to generate Aβ from Wt AβPP [7, 10, 15, 32, 37] which is expressed in the major sporadic population of AD patients. Consideration of CTSB as an alternative Wt β-secretase contributes to the established role of the BACE1 β-secretase [38–40], combined with recently studied proteases with β-secretase activity of meprin [41, 42], delta-secretase [43, 44], and matrix metalloproteinases [45].

## BACE1 ASPARTYL PROTEASE PREFERENTIALLY CLEAVES THE SWE MUTANT β-SECRETASE AβPP SITE, COMPARED TO THE WT β-SECRETASE SITE, FOR Aβ PRODUCTION

Aβ_40_ and Aβ_42_ are known to be produced by the β-secretase BACE1 [33, 46–48]. BACE1 has different proteolytic cleavage capability for Wt versus Swe β-secretase site sequences. The BACE1 protease inefficiently cleaves the WT β-secretase site and efficiently cleaves the Swe β-secretase site [32, 49–51]. Cathepsin B has been postulated as a β-secretase and it differs from BACE1 in cleavage properties since cathepsin B efficiently cleaves the Wt β-secretase site but inefficiently cleaves the Swe β-secretase site [32]. A possible mechanism by which cathepsin B may augment production of Aβ_40_ and Aβ_42_ in models expressing Wt β-secretase activity may involve regulation of BACE1 activity, direct cleavage of the WT β-secretase site, or mechanisms yet to be defined.

Alternatively, cathepsin B may regulate Aβ_40_ and Aβ_42_ production by other means such as lysosomal leakage of cathepsin B to the cytosol to augment the NLRP3 activation of caspase-1 production of pro-inflammatory factor IL-1β [52–54] and to activate cell death through tBid and Bcl-XL regulation [53, 55–57], which thereby regulate production of Aβ_40_ and Aβ_42_ [58, 59].

A distinction between BACE1 and cathepsin B is their role for Aβ production in the constitutive versus regulated secretory pathways of neurons. Neurons possess the regulated secretory pathway that is utilized for activity-dependent secretion of the majority of neurotransmitters [60]. Basal secretion of a small portion of neurotransmitters occurs through the constitutive secretory pathway [60]. BACE1 was identified as a β-secretase for Aβ production through cleavage of the Swe mutant β-secretase site of AβPP that functions in the constitutive secretory pathway of human embryonic kidney cells [49–51]. Cathepsin B was discovered by purification of Wt β-secretase site cleaving activity in regulated secretory vesicles for production of Aβ [61]. These regulated secretory vesicles produce multiple Aβ species of Aβ_40_ and Aβ_42_ as well as the truncated pGlu-Aβ_3 - 40_ and pGlu-Aβ_3 - 42_ [62]. The pGlu-Aβ_3 - 40/42_ peptides accumulate in human AD brains and promote neurotoxicity through oligomerization of Aβ peptides [30, 31, 63].

Significantly, BACE1 does not appear to produce pGlu-Aβ_3 - 40/42_ peptides [10] that are likely the neurotoxic species that promotes oligomerization of Aβ peptides involved in causing AD [30, 31]. However, cathepsin B participates in the production of pGlu-Aβ species in models expressing the WT β-secretase site sequence [10] that is found in most AD patients. While BACE1 inhibitors have been effective in the clinic to reduce Aβ_40_ and Aβ_42_ [64–66], unfortunately, such inhibitors have not significantly improved cognitive deficit of AD [64, 66]. That may be due to BACE1 inhibitors not affecting pGlu-Aβ_3 - 40/42_ production. Immunotherapy by aducanumab targeting Aβ was not efficacious for improving cognition in AD patients [67]. However, donanemab immunotherapy targeting pGlu-Aβ resulted in improved cognition in AD patients [68, 69], showing the importance of pGlu-Aβ in AD. An exciting possibility is that cathepsin B inhibitors may prove useful in the clinic by reducing these pernicious pGlu-Aβ_3 - 40/42_ species of Aβ. More research and development on inhibitors of cathepsin B is warranted.

## CONCLUSION: CTSB PARTICIPATES IN MEMORY DEFICITS AND Wt β-SECRETASE ACTIVITY FOR Aβ PRODUCTION IN HUMAN AβPP MODELS REPRESENTING THE MAJOR SPORADIC AD CONDITION

Evaluation by this review of findings in the literature indicate that elevated CTSB correlates with cognitive deficits in AD patients. In fact, both AD patients and chronic periodontitis-associated AD patients display elevated serum CTSB that correlates with the extent of cognitive deficits [8, 9].

CTSB participates in memory deficits and production of Aβ in AD animal models [7, 10, 15]. Among the six animal models utilized in *CTSB* KO studies (Table 2), the hAβPP-695/Wtβ-Lonγ AD model best represents the majority of the AD population expressing hAβPP-695 as the primary brain AβPP isoform present in neurons. Significantly, *CTSB* KO in the hAβPP-695/Wtβ-Lonγ AD mice results in substantial improvement in memory deficits to nearly normal values, reduced brain levels of Aβ peptides of Aβ_40_, Aβ_42_, pGlu-Aβ_3 - 40_, pGlu-Aβ_3 - 42_, and reduced amyloid plaque load [7, 10]. *CTSB* KO reduces the β-secretase cleavage product of CTFβ generated from hAβPP, suggesting that CTSB participates in Wt β-secretase activity.

*CTSB* KO in mice with the Swe mutant β-secretase of AβPP (hAβPP-695/Sweβ-Lonγ mice) had no effect on memory deficits or Aβ peptides [7]. The Swe mutant hAβPP represents only one AD family [11] and does not represent the major sporadic AD population.

**Fig. 4:**
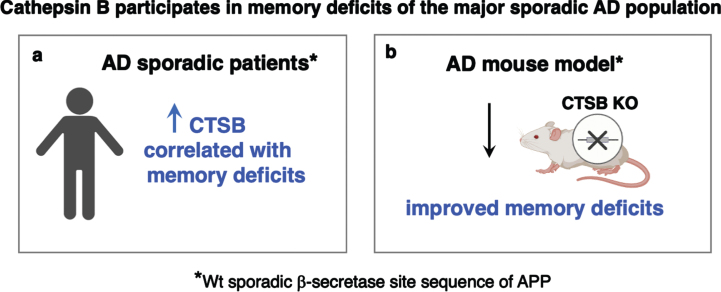
Cathepsin B participates in memory deficits of the major sporadic Alzheimer’s population. (a) CTSB elevation in Alzheimer’s disease (AD) patients correlates with cognitive deficits. Increased levels of CTSB were observed in sporadic AD [1, 2, 4], the major population of AD. Significantly, elevated CTSB was found to be significantly correlated with cognitive decline in AD patients [1]. (b) CTSB gene knockout in animal models of AD results in improved memory deficits. In the AD mouse model expressing hAβPP-695, CTSB gene knockout resulted in substantial improvement in memory deficits [7]. Furthermore, knockout of CTBS in the periodontitis model of AD resulted in improved memory deficits in middle-aged mice [8].

The numerous studies of *CTSB* KO demonstrate that CTSB participates in memory deficits and Aβ production in hAβPP-695 models, combined with clinical data showing correlation of elevated CTSB with cognitive deficits, support the conclusion that CTSB participates in AD memory deficits and pathology. CTSB participates in modulating Wt β-secretase activity for Aβ production in hAβPP models representing the major sporadic AD population (Fig. 4). These findings demonstrate CTSB as a logical drug target for development of therapeutic agents for AD.
